# External signals shape the epigenome

**DOI:** 10.1186/s13059-016-0884-5

**Published:** 2016-02-01

**Authors:** Andreas Lennartsson

**Affiliations:** Department of Biosciences and Nutrition, Karolinska Institute, 141 57 Huddinge, Sweden

## Abstract

A new study shows how a single cytokine, interleukin-4, regulates hematopoietic lineage choice by activating the JAK3–STAT6 pathway, which causes dendritic-cell-specific DNA demethylation.

Please see related Research article: http://www.genomebiology.com/2016/17/1/4

DNA methylation is essential for normal development and cellular homeostasis. Aberrant DNA methylation has been implicated in several cancers and developmental diseases. To elucidate how aberrant DNA methylation patterns contribute to the development of different diseases, it is essential to understand how the DNA methylation machinery regulates the differentiation of normal cells. A new study in *Genome Biology* by Vento-Tormo and colleagues demonstrates how external signals influence epigenetic regulation and cell differentiation [1].

## Interleukin-4 discriminates between two cell-differentiation pathways and induces dendritic cell-specific DNA demethylation

Hematopoiesis is the formation of blood cells, and one of the most extensively characterized differentiation systems, in which hematopoietic stem cells (HSCs) commit to either the lymphoid lineage (and become lymphocytes) or the myeloid lineage (where they can differentiate into cell types including erythrocytes, granulocytes and monocytes). As these cells are short lived and do not self-renew, there needs to be a steady turnover of new blood cells and the maintenance of an HSC pool.

The DNA methylation patterns at promoters and enhancers in different myeloid cell types regulate cell-specific transcriptional activity and thereby contribute to cell identity. However, it is not known how the cell-specific DNA methylation pattern is established. This knowledge is essential to be able to understand how perturbed regulation of DNA methylation contributes to the development of acute myeloid leukemia (AML).

In this new study, Vento-Tormo and colleagues use interleukin-4 (IL-4) to discriminate between the pathways by which human monocytes differentiate into macrophages or dendritic cells (DCs) [1]. They show that the addition of granulocyte-macrophage colony-stimulating factor (GM-CSF) alone causes differentiation of human monocytes into macrophages, whereas GM-SCF and IL-4 in combination lead to differentiation into DCs. The authors show that IL-4 binds to the IL-4 receptor and activates the tyrosine-protein kinase JAK3–STAT6 pathway, which provides a simple membrane-to-nucleus mechanism for rapidly inducing gene expression. This causes activation of the methylcytosine dioxygenase TET2, which has an important regulatory role in the production of blood cells from HSCs and downstream TET2-dependent DC-specific DNA demethylation and gene expression profile. The activation of the signal transducer and activator of transcription STAT6 is probably the key regulator of this process as IL-4 can be bypassed by using constitutively active STAT6 [1].

## TET2 has an important role in myeloid differentiation

An important role for TET2 in late monocytic differentiation has previously been suggested by the authors and by other groups [2]. The entire TET gene family can oxidize methylated cytosine to hydroxymethylated cytosine; however, it is likely that they have partial and non-overlapping target specificities. It has been suggested that TET2 has a stronger effect on enhancer regions than TET1, based on data from mouse embryonic stem cells (ESCs) [3]. TET2 might consequently play an important role in regulating enhancer activity. Indeed, Vento-Tormo and colleagues show that the sites that are demethylated during differentiation to DCs or macrophages are enriched in enhancer regions and are specific for the process [1].

IL-4-induced DNA demethylation, which is mediated by TET2, is an important stage of DC differentiation. TET2 has an important role in myeloid differentiation, and the downregulation of TET2 inhibits both DC and macrophage differentiation as a result of altered demethylation.

TET2-mediated demethylation of DNA is also important for the regulation of HSC proliferation and early lineage choice [4]. TET2-deficient HSCs display reduced DNA hydroxymethylation levels and increased self-renewal. This causes an enlarged HSC and progenitor cell pool owing to increased cell division and self-renewal. Consequently, both the lymphoid and myeloid lineages expand, but with a bias towards the myeloid lineage and development of various myeloid malignancies. However, *TET2* loss-of-function mutations have been observed in both myeloid as well as lymphoid leukemia.

The key role of TET2 in myeloid differentiation is supported by the loss of *Tet2* in an AML mouse model, where it has been shown to result in enhancer hypermethylation. By contrast, hypermethylation was not observed at promoters [5]. These results suggest that TET2 is essential to keep enhancers hypomethylated, which thereby protects the cells from leukemic transformation [5]. *TET2* mutations in AML might therefore disturb the myeloid differentiation program by causing aberrant enhancer activity.

## DNA methylation patterns and cell identity

DNA methylation patterns change during cellular differentiation, which locks the transcriptional state according to the specific cell type. This process occurs at all stages of development, from embryogenesis to adult stem cell differentiation. In a DNA-methylation analysis comparing brain, liver, ESCs and 19 blood and skin cell samples at different stages of maturation, it was shown that each cell type can be distinguished based on its DNA methylation pattern [6]. This analysis shows that different cell types are defined by unique DNA methylation patterns.

Vento-Tormo and colleagues found that the main changes in DNA methylation occur during differentiation, and only very few changes take place when DCs or macrophages become activated with bacteria-derived lipopolysaccharide (LPS). By contrast, thousands of genes change expression in both the differentiation and activation processes [1]. This suggests that, when the cell identity is established, only minor changes in DNA methylation occur. This is in agreement with DNA methylation as a mechanism for a cell to “remember” its identity and transcriptional program during development. This has previously also been shown in another myeloid lineage, the neutrophil lineage. In neutrophil differentiation, DNA demethylation predominantly occurs before the pro-myelocytic stage of differentiation [7]. At this stage, all lineage choices are made and the cell fate established, and therefore no changes in DNA methylation are required.

DNA methylation represses transcription through either active transcriptional silencing or by inhibiting transcriptional activation, depending on the precise genomic location and context of the methylated cytosines. The majority of cytosines that are methylated during differentiation are already transcriptionally silent in ESCs [8], suggesting that silencing precedes DNA methylation. In a similar, but opposite, manner, Vento-Tormo and colleagues show that several genes demethylated during DC or macrophage differentiation are not expressed until the macrophage becomes activated by LPS. Hence, DNA demethylation might not directly regulate transcription, but instead creates a permissive chromatin state that can be activated upon stimulation.

Interestingly, IL-4 signaling not only induces DC-specific DNA demethylation but it also prevents demethylation of cytosines during macrophage differentiation [1]. A similar mechanism has been described in lymphoid differentiation, where lymphoid progenitors have enhanced methylation at binding sites of myeloid transcription factors [6]. The increased methylation might inhibit the binding of these factors and block myeloid differentiation in lymphoid cells, in a manner similar to that of the IL-4-induced inhibition of demethylation at macrophage-specific sites. Therefore, DNA methylation defines cell identity by allowing one cell identity, while blocking the other.

## Aberrant DNA methylation in diseases

Epigenetic alterations can occur either before, or as a consequence of, somatic mutations. Somatic mutations in the DNA methylation machinery are documented in several cancers. In acute myeloid leukemia (AML), mutations in DNA methylation regulators such as *DNMT3A*, *TET2*, *IDH1* and *IDH2* are frequent, and loss of function of *TET2* and *DNMT3A* are early events in leukemogenesis [9].

By contrast, cases of aberrant epigenetic landscapes that do not have co-current somatic mutations have proven difficult to find. However, childhood brain tumors, ependymomas, display the CpG island methylator phenotype (CIMP), which defines cancers with a high degree of CpG island methylation, in combination with extremely low mutation levels and no somatic single-nucleotide variants [10]. The characteristics of ependymomas support the model that epigenetic aberrations can occur without any preceding genetic mutations. These epigenetic aberrations are likely to be created owing to modified external signals. Aberrant epigenetic patterns are also found in many non-cancer diseases, where the environment and external signals are major contributors to the disease. Therefore, knowledge of how external signals at different levels affect the DNA methylation pattern is crucial to understand the development of the affected disease, including AML.

## Concluding remarks

The cross-talk between transcription factors, such as STAT6, and regulators of DNA methylation, such as TET2, facilitates a greater control of transcriptional regulation and stability of cell identity than would be possible if each acted separately. Vento-Tormo and colleagues demonstrate how external signaling mediated by IL-4 contributes to these interactions, which in turn regulate DC differentiation. The instructing role of cytokines in hematopoiesis is well established, but the authors have demonstrated the detailed molecular interplay between cytokine signaling pathways and epigenetic mechanisms, which has not previously been shown.

## Abbreviations

AML: Acute myeloid leukemia
CIMP: CpG island methylator phenotype
DC: Dendritic cell
ESC: Embryonic stem cell
GM-CSF: Granulocyte-macrophage colony-stimulating factor
HSC: Hematopoietic stem cell
IL-4: Interleukin-4
LPS: Lipopolysaccharide.
